# An exploratory clinical trial of apatinib combined with intensity‐modulated radiation therapy for patients with unresectable hepatocellular carcinoma

**DOI:** 10.1002/cam4.4900

**Published:** 2022-05-27

**Authors:** Hu Qiu, Shaobo Ke, Gaoke Cai, Yong Wu, Jin Wang, Wei Shi, Jiamei Chen, Jin Peng, Baoping Yu, Yongshun Chen

**Affiliations:** ^1^ Department of Clinical Oncology Renmin Hospital of Wuhan University Wuhan China; ^2^ Department of Radiation and Medical Oncology Zhongnan Hospital of Wuhan University Wuhan China; ^3^ Department of Gastroenterology Renmin Hospital of Wuhan University Wuhan China

**Keywords:** apatinib, hepatocellular carcinoma, radiation therapy, vascular endothelial growth factor

## Abstract

**Purpose:**

To evaluate the clinical efficacy and safety of apatinib combined with intensity‐modulated radiation therapy (IMRT) in patients with unresectable hepatocellular carcinoma (uHCC).

**Materials and methods:**

Open‐label, single‐arm, exploratory clinical trial of apatinib combined with IMRT for uHCC patients. Patients aged 18–75 years with adequate hematological, liver, and renal functions and Eastern Cooperative Oncology Group (ECOG) performance status of ≤2 were enrolled in this study from March 2017 to September 2020. Patients were received IMRT (biological effective dose: 46–60 Gy) and continuous apatinib (250–500 mg/day) oral administration until HCC progression or unacceptable toxic effects. The endpoints included progression‐free survival (PFS), overall survival (OS), disease control rate (DCR), objective response rate (ORR), and safety. The trial registration number is ChiCTR‐OPC‐17011890.

**Results:**

A total of 33 patients have taken part in the study. The median age was 58 years old (range 32–77), 27 (81.9%) patients were ECOG PS 0–1, and 28 (84.9%) patients were male. In addition, 25 (75.7%) patients suffered from hepatitis B, 32 cases (97.0%) were in Barcelona Clinic Liver Cancer (BCLC) Stages B–C, and eight (24.2%) had portal vein involvement. Moreover, 12 (36.4%) and 21 (63.6%) patients received apatinib as first‐line and second or later‐line therapy, respectively. The average follow‐up was 11.4 months, the median PFS was 7.8 months (95% confidence interval: 3.9–11.7). The OS rates at 6 and 12 months were 96.7% and 66.2%. The ORR and DCR were 15.1% and 81.8%, respectively. Hepatic toxicity was the most common treatment‐related adverse events in Grades 3–4 (12.1%). No radiation‐induced liver disease and Grade 5 toxicity were recorded.

**Conclusion:**

Apatinib combined with IMRT is a safe and effective method to improve PFS and DCR and has good anti‐tumor activity in patients with uHCC.

## Graphical abstract

An open‐label, single‐arm, exploratory clinical trial of apatinib combined with IMRT is a safe and effective method to improve PFS and DCR and has good anti‐tumor activity in patients with unresectable HCC. The median PFS was 7.8 months. The OS rates at 6 months and 12 months were 96.7% and 66.2%. The ORR and DCR were 15.1% and 81.8%, respectively. No radiation‐induced liver disease and grade 5 toxicity were recorded.

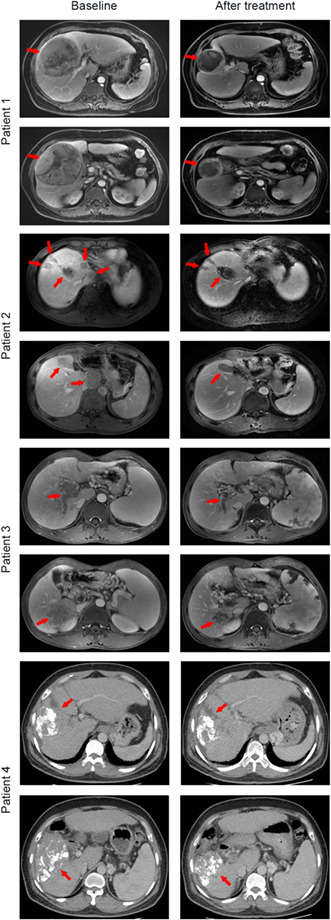

## 1 INTRODUCTION

Primary liver cancer is one of the most common malignant tumors all over the world, with high morbidity and mortality. In 2020, about 910,000 new patients with primary liver cancer and 830,000 cases were dead in this disease worldwide, and approximately 45.1% of these new cases and 47.0% of these deaths were expected to occur in China.^1^ Hepatocellular carcinoma (HCC) is a particular tissue subtype of primary liver carcinoma accounting for about 85%–90% of cases, while intrahepatic cholangiocarcinoma (ICC) and mixed type of HCC‐ICC are the other subtypes.^2^ In general, the prognosis of primary liver cancer is poor. The morbidity/mortality ratio of HCC is 1:0.9, and the 5‐year survival rate is 15%–19% in North America and only 12.1% in China.^3^, ^4^, ^5^, ^6^

Vascular endothelial growth factor (VEGF) plays an integral role in tumor growth and angiogenesis, and anti‐angiogenic therapy plays an essential role in tumor therapy.^7^ Anti‐angiogenesis drugs have been widely used in non‐small cell lung cancer,^8^, ^9^ esophageal cancer^10^, ^11^ and HCC,^12^, ^13^, ^14^ and the main indication group is patients with recurrence and metastasis. Apatinib, a potent small‐molecule tyrosine kinase inhibitor (TKI), is a high selectivity inhibitor of the VEGF receptor‐2 (VEGFR2).^15^ In the AHELP study,^16^ apatinib monotherapy was administered to second‐line or later treatment for patients with advanced HCC. The objective response rate (ORR) and disease control rate (DCR) were 11% and 61%, respectively. The median progression‐free survival (PFS) and overall survival (OS) were 4.5 and 8.7 months, respectively. The main application mode is anti‐angiogenesis combined with chemotherapy, targeted therapy or immunotherapy, and single‐drug anti‐angiogenesis is only recommended for second or later‐line therapy. Several studies have reported that apatinib combined with transarterial chemoembolization (TACE) and programmed death‐ligand 1 showed potent anti‐tumor activities and tolerable toxicities in advanced HCC.^17^, ^18^, ^19^

As an effective antitumor therapy, radiotherapy has been shown to have the following effects in HCC: Local tumor control, tumor vessel inhibition, and stimulation of immune cell aggregation.^20^ From the perspective of mechanism, anti‐angiogenesis drugs have the effect of “pruning” abnormal blood vessels, which may improve tumor perfusion/oxygenation, and thus have the potential for radiotherapy synergism.^21^ At present only less sample size of the low level of clinical trials in exploring the effect of radiotherapy with targeted or immune therapy in HCC. Although anti‐angiogenesis therapy can extend the PFS and OS in patients with advanced HCC,^12^, ^14^ SBRT in combination with sorafenib therapy of HCC I phase of clinical trial results show that combination therapy is not the same as imagination to improve the effect.^22^ Instead, the increase in adverse events (AEs), especially RILDs, suggests caution in subsequent clinical trials. Herein, we conducted an exploratory clinical trial to evaluate the efficacy and safety of apatinib in combination with intensity‐modulated radiation therapy (IMRT) for unresectable HCC (uHCC).

## 2 MATERIALS AND METHODS

### 2.1 Trial design and patient enrollment

This study was an open‐label, single‐arm, exploratory clinical trial (Identification number: ChiCTR‐OPC‐17011890), registered in the China clinical trial registration center and approved by the ethics committee of the people's Hospital of Wuhan University. In accordance with the Declaration of Helsinki, all patients receive informed consent before undergoing any particular research procedure.

Eligible patients were aged 18–75 years, and the diagnosis was confirmed histologically or cytologically or identified clinically according to the standards of the American Association for the Study of Liver Disease. Patients had unresectable disease or relapse/metastasis after receiving systematic treatments. Other inclusion criteria were at least one measurable target lesions according to the Response Evaluation Criteria in Solid Tumors (RECIST) 1.1; treatment discontinuation of the last previous systemic therapy at least 2 weeks before enrollment and all AEs relieved to grade 1 or lower according to Common Toxicity Criteria for Adverse Events (CTVAE) version 5.0; the Eastern Cooperative Oncology Group (ECOG) performance status score ≤2, the A or B classification on the Child‐Pugh liver function scale, and Barcelona Clinic Liver Cancer (BCLC) Stage A, B or C; a life expectancy of at least 12 weeks. In addition, all eligible patients had adequate hematological and organ (liver, renal, and pancreatic) function and did not include RT and apatinib in prior liver treatments.

Critical exclusion criteria were as follows: History of autoimmune disease, active hemorrhage within 2 months, deficient organ function or other uncontrolled systemic diseases, esophageal or gastric varices bleeding or high risk on bleeding without treatment or incompletely treatment, and mean liver dose exceeding 25 Gy.

### 2.2 Procedures

All enrolled patients received continuous apatinib (250–500 mg/day) orally in combination with IMRT (biological effective dose: 46–60 Gy) and underwent apatinib dose modifications followed by reductions for related toxicities. For therapy planning purposes, all patients were undergone contrast enhanced computed tomography (CT) simulation. Gross tumor volume (GTV) and planning target volume (PTV) were determined by magnetic resonance imaging (MRI) or CT. An internal target volume (ITV) was generated to encompass the range of breathing motion for patients with free‐breathing. GTV or ITV was a standard margin of 5 mm axially and 8 mm superiorly and inferiorly was added for the PTV. Radiation fields included primary tumor and portal vein tumor thrombosis (PVTT) as they were visualized on the planning CT or after fusion with MRI. The IMRT schedule is delivered in 2.0–3.5 Gy per fraction and five fractions per week. The organs at risk constraints are listed as follows: Spinal cord <45 Gy, kidney V20 <30%, stomach V40 <30%, small intestine V40 <1 cc and mean liver doses <25 Gy.

### 2.3 Endpoints and assessments

The primary endpoint was PFS, measured from start point of the treatment until the date of first documented disease progression or date of death from any cause. The secondary endpoints included OS (defined as the first time from start point of the treatment to death or the last follow‐up), DCR, ORR, and safety. CT or MRI‐based tumor assessment was conducted before treatment and every 6–8 weeks after treatment until disease progression or death. ORR and DCR were evaluated according to the RECIST 1.1. Safety assessments included monitoring and recording of AEs, which were assessed in accordance with the CTVAE version 5.0.

### 2.4 Statistical analysis

Summary statistics for normally distributed quantitative variables were expressed as means and standard deviations. For non‐normallydistributed variables were used median; categorical data were presented by percentages. ORR and DCR were calculated with 95% CI using the Clopper Pearson method. Kaplan–Meier method was used for the analysis of PFS and OS. Significant differences were determined by the log‐rank test. A two‐sided *p*‐value less than 0.05 was considered to be statistically significant. All statistical tests were used by SPSS version 20.0. The survival curve was made using the GraphPad Prism software version 7.0.

## 3 RESULTS

### 3.1 Patients

A number of 33 patients in uHCC were recruited in the intention‐to‐treat population and received apatinib combined with IMRT between March 1, 2017, and September 1, 2020. The median age in the cohort was 58 years old (range: 32–77). 84.9% patients were male (28/33), ECOG PS of 0–1 score (27/33, 81.8%), Child‐Pugh class A (25/33, 75.8%), AFP level ≥400 ng/ml (22/33, 66.7%) and associated with hepatitis at baseline. BCLC stage B was noted in 51.5% of patients and stage C was in 45.5%. Meanwhile, 36.4% and 63.6% of patients received apatinib as first‐line and second or later‐line treatment, respectively. Among 21 patients receiving second or later‐line treatment, 15 patients only accepted sorafenib or chemotherapy as first‐line treatment, and six patients received regofinib as second‐line treatment after sorafenib or chemotherapy treatment. The percent of patients with portal vein involvement and extrahepatic metastasis at baseline were 24.2% and 21.2%, respectively. The median tumor size was 80 mm for all patients (Table 1).

**TABLE 1:** Clinical baseline characteristics of unresectable advanced HCC patients

Characteristics	Patients
Sex (*n*, %)	
Male	28 (84.8)
Female	5 (15.2)
Age (years)	
Mean ± *SD*	56.7 ± 10.1
Median (range)	58 (32–77)
ECOG performance status score (*n*, %)	
0	2 (6.1)
1	25 (75.7)
2	6 (18.2)
Child‐Pugh class (*n*, %)	
A	25 (75.7)
B	6 (18.2)
C	2 (6.1)
Hepatitis (*n*, %)	
Yes	25 (75.7)
No	8 (24.2)
AFP	
<400 ng/ml	21 (63.6)
≥400 ng/ml	12 (36.4)
BCLC stage (*n*, %)	
A	1 (3.0)
B	18 (54.6)
C	14 (42.4)
Prior systemic treatment regiments (*n*, %)	
1	12 (36.4)
≥2	21 (63.6)
Portal vein involvement (*n*, %)	
Yes	8 (24.2)
No	25 (75.7)
Extrahepatic metastasis (*n*, %)	
Yes	7 (21.2)
No	26 (78.8)
Sites of extrahepatic (*n*, %)^a^	
Lung	4 (12.1)
Bone	3 (9.1)
Adrenal	1 (3.0)
Lymph node	1 (3.0)
Tumor size (mm)	
Mean ± *SD*	78.1 ± 52.6
Median (range)	80 (7–203)

Abbreviations: AFP, α‐fetoprotein; BCLC, Barcelona Clinic Liver Cancer; ECOG PS, Eastern Cooperative Oncology Group; *SD*, standard deviation.

^a^ Two patients in HCC with lung metastases had bone metastases.

### 3.2 Efficacy

**FIGURE 1:**
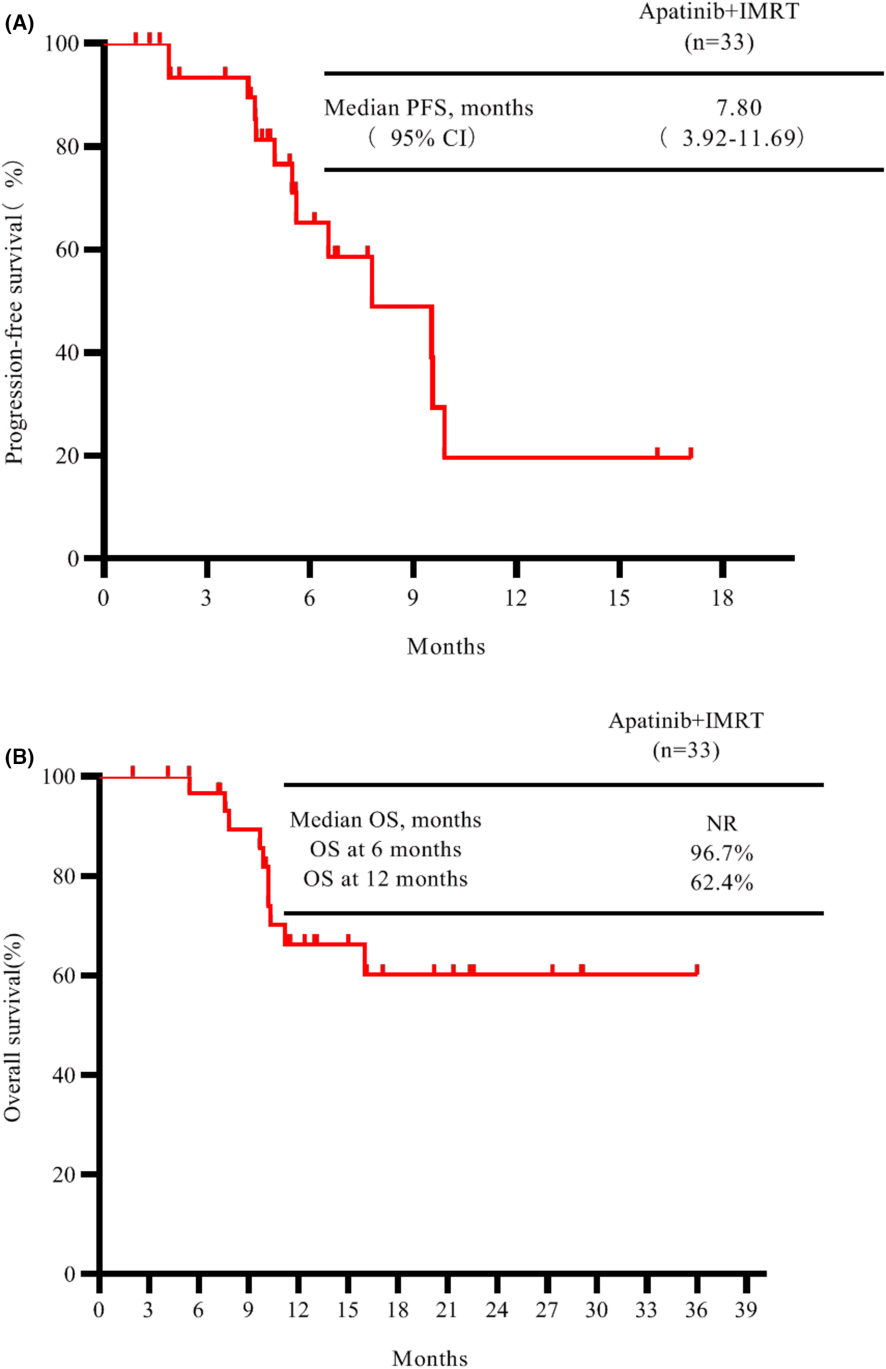
Kaplan–Meier analysis of PFS (A) and OS (B) in patients with unresectable hepatocellular carcinoma or relapse/metastasis after receiving systematic treatments. IMRT, intensity‐modulated radiation therapy; OS, overall survival; PFS, progression‐free survival.

**FIGURE 2:**
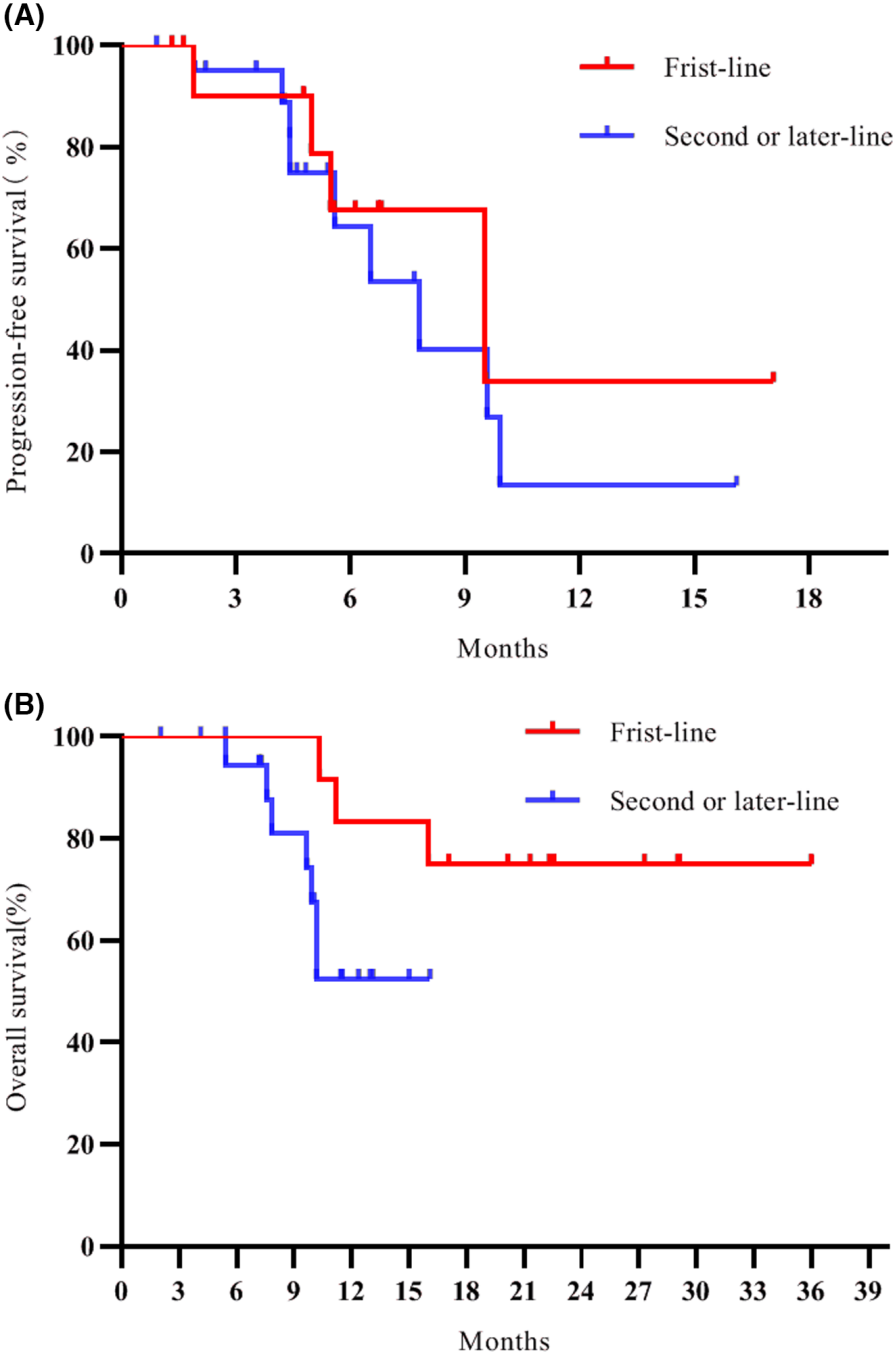
Progression‐free survival (A) and overall survival (B) of patients in first‐line and second or later‐line groups.

**FIGURE 3:**
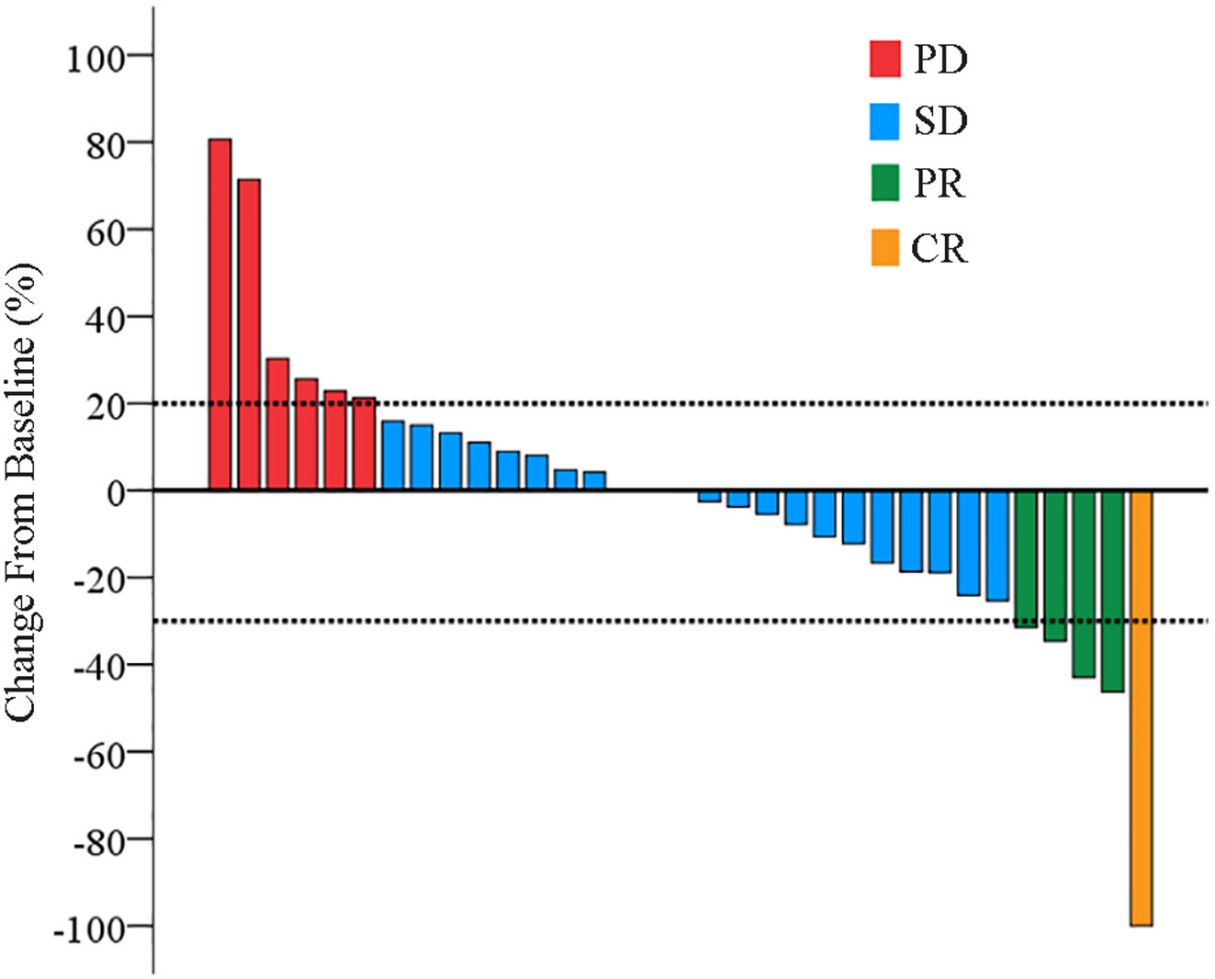
Waterfall plot for the best percentage change in target lesion size. Waterfall plot for the best percentage change in target lesion size is shown for 33 patients who had unresectable hepatocellular carcinoma or relapse/metastasis after receiving systematic treatments. The color indicates the type of response. Red, blue, green, and yellow represents progressive disease (PD), stable disease (SD), partial response (PR), and complete response (CR), respectively. Two patients had 0% change from baseline. The dotted line at 20% represents the boundary for PD, and the dotted line at −30% represents the boundary for PR.

**FIGURE 4:**
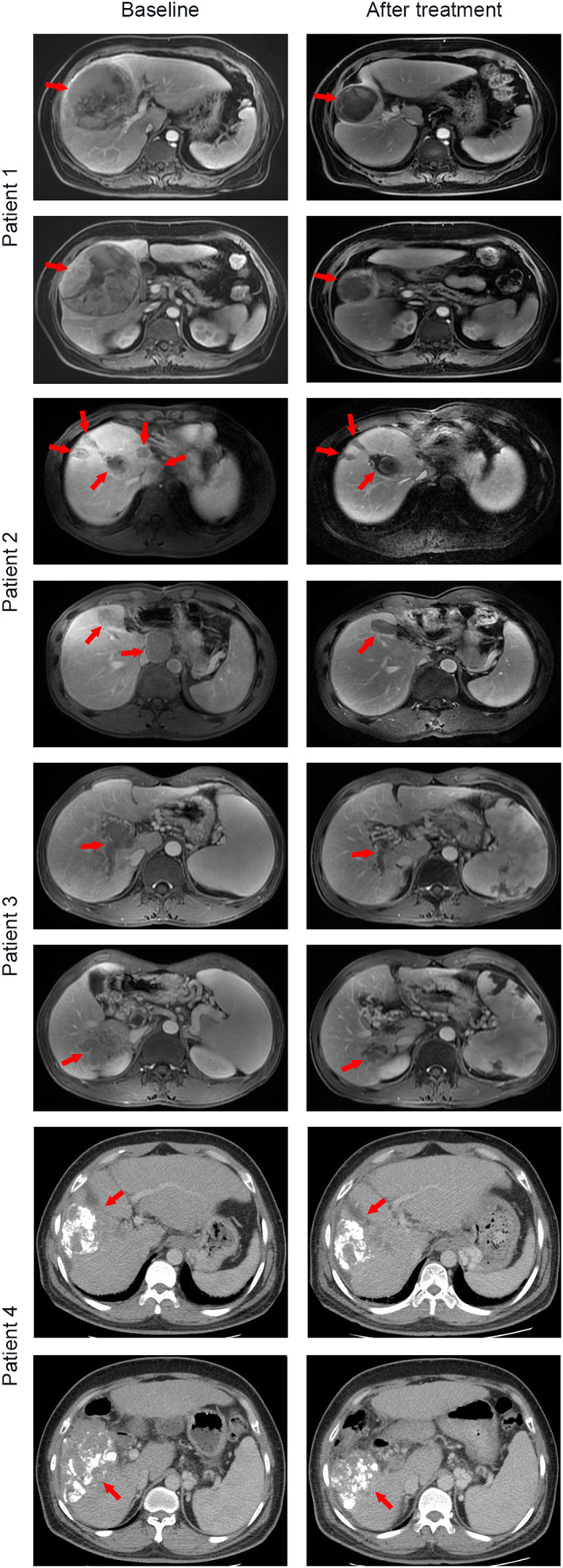
Magnetic resonance imaging (MRI) or computed tomography (CT) scans of the four responders with partial response. The four responders with partial response had more than 30% shrinkage of their tumors by MRI or CT scans. Of the four responders with their characteristics, Patient 1 had a large liver cancer with the residual liver function of less than 30% and was inoperable, Patient 2 had recurrence and metastasis after surgical treatment, Patient 3 had portal venous thrombosis, which was initially unresectable and Patient 4 had progressed after multiple interventions.

As of the cut‐off date (February 1, 2021), the median follow‐up time for this intended treatment population was 11.4 months. The median PFS was 7.8 months (95% CI: 3.9–11.7) (Figure 1A). The estimated survival rates at 6 and 12 months respectively were 96.7% and 66.2% (Figure 1B). In subgroup analysis, the median PFS was 9.5 months in first‐line group, as compared with 7.8 months in second or later‐line group (hazard ratio for progression or death in first‐line group, 0.72; 95% CI: 0.23–2.12) (Figure 2A). The 12 months of OS rates was 75% in first‐line group, significantly better than the second or later‐line group (hazard ratio for death in first‐line group, 0.33; 95% CI: 0.09–1.15) (Figure 2B). Additionally, ORR was tested after significant improvement for PFS. The confirmed ORR was 15.1%, one (3.0%) patient had a complete response according to RECIST 1.1 (Table 2). The DCR was 81.8% (objective response plus stable disease [SD]). At the cutoff date, six (18.2%) patients have had disease progression or dead. Moreover, 27 patients were without disease progression, of whom 22 (66.7%) had SD. Tumor shrinkage occurred in 31 of 33 patients, and at least one post‐baseline efficacy evaluation was performed (Figure 3). Compared with baseline, the average optimal percentage change in target lesion size was −2.1%. The four responders with PR had more than 30% shrinkage of their tumors by MRI or CT scans (Figure 4). Of the four responders with their characteristics, Patient 1 had a large liver cancer with the residual liver function of less than 30% and was inoperable, Patient 2 had recurrence and metastasis after surgical treatment, Patient 3 had portal venous thrombosis, which was initially unresectable and Patient 4 had progressed after multiple interventions.

**TABLE 2:** Secondary efficacy outcomes

Variable	Apatinib‐IMRT (*n* = 33)
Best over response (*n*, %)	
Complete response	1 (3.0)
Partial response	4 (12.1)
Stable disease	22 (66.7)
Progressive disease	6 (18.2)
Objective response rate (% [95% CI])	15.1 (2.2–28.1)
Disease control rate (% [95% CI])	81.8 (67.9–95.7)
OS rate (%)	
6 months	96.7
12 months	66.2

Abbreviation: IMRT, intensity‐modulated radiation therapy.

### 3.3 Safety

Safety analyses were included in all patients who received IMRT combined with continuous apatinib oral administration. The most frequent treatment‐related AEs are summarized in Table 3. The most common any‐grade treatment‐related AEs were anorexia (*n* = 14, 42.4%) and anemia (*n* = 14, 42.4%). In this trial, the incidences of hypertension, proteinuria, and hand‐foot syndrome were the most common AEs of apatinib, which were 21.2%, 3.0% and 12.1%, respectively. The treatment‐related hematological AEs were anemia (42.4%), thrombocytopenia (36.4%), and leukopenia (30.3%), respectively. The most common treatment‐related hepatotoxic AEs were increased in blood bilirubin (39.4%), γ‐glutamyl transferase (36.4%), alanine aminotransferase (30.3%), and alkaline phosphatase (27.3%). Additionally, hepatic toxicity (12.1%), hypertension (9.1%), hand‐foot syndrome (6.1%), and anemia (6.1%) were the most common treatment‐related AEs in the Grades 3–4. No serious AEs and drug or radiotherapy‐related deaths occurred in this clinical trial. All patients had finished the entire treatment course, and no patient discontinued any treatment because of AEs. AEs leading to dose modification of apatinib occurred in 12.1% of patients. No patient needed dose modification of IMRT.

**TABLE 3:** Most common treatment‐related adverse events (AEs) in patients receiving apatinib plus intensity‐modulated radiation therapy (*N* = 33)

AE	Number of patients (%)			
	Any grade	Grade 1	Grade 2	Grade 3 or 4
Anemia	14 (42.4)	3 (9.1)	9 (27.3)	2 (6.1)
Thrombocytopenia	12 (36.4)	2 (6.1)	9 (21.2)	1 (3.0)
Leukopenia	10 (30.3)	0	10 (30.3)	0
Alanine aminotransferase increased	10 (30.3)	4 (12.1)	5 (15.2)	1 (3.0)
Aspartate aminotransferase increased	1 (3.0)	1 (3.0)	0	0
Blood bilirubin increased	13 (39.4)	4 (12.1)	9 (27.3)	0
Alkaline phosphatase increased	9 (27.3)	2 (6.1)	7 (21.2)	0
γ‐glutamyl transferase increased	12 (36.4)	0	9 (27.3)	3 (9.1)
Hand‐foot syndrome	4 (12.1)	1 (3.0)	1 (3.0)	2 (6.1)
Hypertension	7 (21.2)	1 (3.0)	3 (9.1)	3 (9.1)
Diarrhea	4 (12.1)	2 (6.1)	1 (3.0)	1 (3.0)
Anorexia	14 (42.4)	5 (15.2)	8 (24.2)	1 (3.0)
Nausea and vomiting	10 (30.3)	4 (12.1)	5 (15.2)	1 (3.0)
Gastrointestinal hemorrhage	2 (6.1)	0	2 (6.1)	0
Abdominal distension	7 (21.2)	1 (3.0)	6 (18.2)	0
Weight decreased	12 (36.4)	7 (21.2)	5 (15.2)	0
Fatigue	5 (15.2)	0	5 (15.2)	0
Fever	4 (12.1)	2 (3.0)	2 (3.0)	0
Proteinuria	2 (3.0)	0	2 (3.0)	0

## 4 DISCUSSION

To the best of our knowledge, it is the first time to explore the effect of VEGFR‐2 inhibitors in combination with IMRT in patients with unresectable advanced HCC. This study has shown that apatinib combined with IMRT achieved an ORR of 15.1%, and one patient achieved a complete response. Furthermore, 66.7% of the patients were SD, and the DCR was as high as 81.8%. The median follow‐up time was 11.4 months, besides, the median PFS was 7.8 months. The 6‐month OS rate was 96.7% and the 12‐month OS rate was 66.2%. In addition, combination treatment on apatinib and IMRT was confirmed to have controllable toxicity characteristics.

Hepatocellular carcinoma is a highly vascularized neoplasm due to the active formation of neovascularization that grow and proliferate.^23^ It has a strong tendency to invade surrounding hepatic blood vessels, and clinical data showed that invasion of the portal vein and its branches existed in about 31.4%–34% of HCC patients.^24^ In the present study, the portal vein and its branches were invaded in 24.2% of unresectable advanced HCC patients. For the past 15 years, the treatment of HCC has been dominated by multi‐target TKIs or single‐target drugs against angiogenesis. To date, only three targeted therapies have been approved in China: Sorafenib and lenvatinib for first‐line treatment and regorafenib for second‐line treatment. SHARP, ORIENTAL, and REFLECT studies confirmed that sorafenib and lenvatinib have significant efficacy in the first‐line regimen of advanced HCC.^12^, ^14^, ^25^ In the RESOURCE study for second‐line treatment of advanced HCC, the median OS in the regorafenib group and the placebo group was 10.6 and 7.8 months, respectively. The median PFS was 3.1 and 1.5 months, respectively, and the effective rates were 10.6% and 4.1%, respectively.^26^ Apatinib is a novel small molecule TKI, whose main target is VEGF‐2. AHELP study of second‐line treatment of advanced HCC with apatinib was presented at the 2020 ASCO Annual Meeting. Patients were randomly divided into apatinib 750 mg or placebo group, with a median OS of 8.7 and 6.8 months, respectively (*p* = 0.0476).^16^ The median PFS was 4.5 and 1.9 months (*p* < 0.0001), and the effective rates were 10.7% and 1.5%, respectively. Although these studies have shown that anti‐angiogenic treatment is effective for advanced HCC, the PFS is unsatisfactory, and the effective rate is only around 10%, mainly due to the poor local control rate. Hence, combined local treatment is required. In this study, a higher proportion of patients had ECOG PS of 1–2 than in the SHARP, ORIENTAL, and REFLECT studies (94% in this study, 46% in SHARP, 75.6% in ORIENTAL, and 36% in REFLECT). Additionally, this study included patients who had been refractory with postoperative progression after surgery or interventional therapy, including those who had been treated with second or later‐line of previous systemic treatment regimens (63.6%), whereas the SHARP, ORIENTAL, and REFLECT studies enrolled patients who had not received previous systemic therapy. However, the present study has shown that a median PFS was 7.8 months, besides, the OS rates at 6‐ and 12‐month respectively were 96.7% and 66.2%, indicating that the survival benefit was significantly better than that reported in previous studies.^9^, ^10^, ^12^, ^16^ Although this was an exploratory study with small sample size, it showed that the novel treatment modality of apatinib combined with IMRT provides better survival benefits for patients with uHCC.

Mechanistically, the normalization of dysfunctional tumor vascular system by a reasonable dose of antiangiogenic drugs may improve the tumor perfusion and oxygenation.^27^ Additionally, VEGF‐targeted therapy can revert the immunosuppressive effects of VEGF. For example, VEGF can inhibit the function and maturation of dendritic cells,^28^, ^29^ as well as the infiltration and function of cytotoxic T lymphocytes.^30^ As an effective means of tumor treatment, radiotherapy has been shown to have the following effects in HCC: Permanent control of tumor,^31^ inhibition of tumor blood vessels, and stimulation of immune cell aggregation.^32^ Therefore, radiotherapy has a potential synergistic effect. The results of a previous phase II study showed that the ORR at 1 month after radiotherapy was 55%, the 2‐year OS rate and the PFS rate in the irradiated field were 32% and 39%, respectively.^33^ However, the incidence of grade ≥2 hepatotoxicities reached 35%, including three deaths, suggesting a greater potential risk of concurrent therapy. Another retrospective analysis found that although the addition of sorafenib to radiotherapy alone in addition to TACE therapy prolonged PFS, the incidence of grade 3 TRAE was significantly higher.^34^ Thus, strategies to reduce toxicity from combined modality therapy with an anti‐angiogenesis drug are essential. Although a meta‐analysis showed that a dose of 750–850 mg apatinib resulted in a higher ORR and DCR,^35^ the side effect of radiation‐induced liver disease (RILD) remains a significant issue when planning radiotherapy for HCC. Therefore, we adopted 250–500 mg once daily as the apatinib initial dose. In this study, 39.4% of patients receiving hepatic IMRT with a concurrent regimen of 500 mg apatinib demonstrated acceptable tolerability, and 12.1% of patients could not tolerate the gastrointestinal toxicities, resulting in the dose reduction to 250 mg. Thus, the well‐known toxicities of apatinib did not appear to increase during IMRT with the minimum dose 46 Gy, even though 21.2% of patients were Child‐Pugh B‐C. Four patients (12.1%) developed treatment‐related hepatic toxicity grade 3–4, but liver function recovered to normal after dose adjustment. No RILD was observed. Additionally, the DCR reached 81.8%and the ORR reached 15.2%. Therefore, we recommend that the initial dose for apatinib should be 500 mg, alternating to 250 mg if associated toxicity occurs.

Hypertension, proteinuria and hand‐foot syndrome were the most common apatinib related AEs.^17^, ^35^, ^36^, ^37^ In this trial, the incidences of hypertension, proteinuria, and hand‐foot syndrome were 21.2%, 3.0%, and 12.1%, respectively, which were generally lower than the previous studies, which may be related to the lower dosage of apatinib used in our trial. In addition, the common AEs caused by radiotherapy mainly include fatigue, recurrent vomiting, bone marrow suppression, liver function damage, and upper gastrointestinal bleeding in severe cases.^33^, ^34^ The combination of apatinib and radiotherapy inevitably increases these adverse reactions. In the safety investigation of this study, the treatment‐related hematological AEs were anemia (42.4%), thrombocytopenia (36.4%), and leukopenia (30.3%). Increases in blood bilirubin (39.4%), γ‐glutamyl transferase (36.4%), alanine aminotransferase (30.3%), and alkaline phosphatase (27.3%)were all the most common treatment‐related hepatotoxic AEs. Hematological toxicity was generally consistent with previous studies,^35^ but hepatotoxicity was lower. Additionally, the most common treatment‐related AEs of grades 3–4 were hepatic toxicity (12.1%), hypertension (9.1%), hand‐foot syndrome (6.1%), and anemia (6.1%). No RILD and grade 5 toxicity were recorded.

This exploratory clinical study had some limitations. First, it was a single‐arm clinical study with a limited sample size, and some selection bias may have existed. In addition, several randomized controlled studies including IMBRAVE‐150, KEYNOTE‐524, CHECKMATE‐040, CHECKMATE‐459, and KEYNOTE‐224^38^, ^39^, ^40^, ^41^, ^42^ have established the combination of anti‐angiogenic therapy and immunotherapy as the standard first and second‐line treatments for advanced HCC. However, based on socioeconomic status and systemic status assessments, apatinib combined with radiotherapy is only appropriate for patients who are not suitable for immunotherapy. Notably, IMBRAVE‐150 study demonstrated that anti‐angiogenic therapy combined with immunotherapy is an effective first‐line standard regimen for HCC. A previous phase II clinical study showed that sorafenib combined with external radiotherapy did not improve the efficacy, but increased the toxic and side effects, suggesting that sorafenib must be cautiously used in combination with radiotherapy for intracerebral lesions.^33^ However, with the continuous progress of radiotherapy and extensive application of 3D‐CRT, IMRT, and IGRT, accurate precision radiotherapy has been established in clinical practice, and a large‐population‐based study should be conducted to validate the efficacy of apatinib combined with IMRT in different subgroups of uHCC.

In summary, this exploratory clinical study showed an improved PFS and DCR in patients with unresectable advanced HCC. The safety of apatinib combined with IMRT was consistent with the known safety of anti‐angiogenic agents and radiotherapy in HCC, and no new safety indicators were found. Therefore, apatinib combined with IMRT may be a new treatment modality for unresectable advanced HCC.

## AUTHOR CONTRIBUTIONS

Hu Qiu and Shaobo Ke have the same contribution to this work. Hu Qiu and Shaobo Ke analyzed the data and wrote a paper. Yongshun Chen and Baoping Yu applied for the funding and designed the clinical trial. Gaoke Cai, Yong Wu, Jin Wang, Wei Shi, Jiamei Chen, and Jin Peng performed the trial. Yongshun Chen edited the paper. All authors contributed to the article and approved the summited version.

## FUNDING INFORMATION

This work was supported by the Central Leading Local Science and Technology Development Special Foundation (ZYYD2020000169). No benefits in any form have been or will be received from a commercial party related to the subject of this work.

## CONFLICT OF INTEREST

The authors declare that there is no conflict of interest.

## ETHICS APPROVAL AND CONSENT TO PARTICIPATE

The institutional review board and ethics committee of Renmin Hospital of Wuhan University approved the present study, and informed consent was confirmed by the participator.

## CLINICAL TRIAL REGISTRATION NUMBER

The trial registration number is ChiCTR‐OPC‐17011890.

## Data Availability

No applicable.
